# A dam or a polder? Stakeholders’ dispute over the “right” flood-protection measure in the Czech Republic

**DOI:** 10.1007/s13280-024-02022-7

**Published:** 2024-04-26

**Authors:** Ivan Andráško, Barbora Duží, Stanislav Martinát

**Affiliations:** Department of Environmental Geography, Institute of Geonics of the Czech Academy of Sciences, Drobného 28, 602 00 Brno, Czech Republic

**Keywords:** Czech Republic, Dispute/conflict, Flood-protection measures, Interviews, Polder, Stakeholder involvement

## Abstract

This study focuses on the Skalička Waterwork (SWW), a largely debated and media-covered water-related/flood-protection project in the Czech Republic. Relying primarily on stakeholder interviews, we traced back and reconstructed the project’s development, including its key tipping points reflecting the changing societal preferences for particular measures, yet also the involvement of individual actors/stakeholders, and their differing views. The case eventually crystallized into the “dam versus polder” dispute; concerned by the repercussions for the local landscape, a joint initiative of NGOs, local activists, and politicians not only opposed the dam variant proposed by the state river basin administration but also succeeded in pushing through the alternative scheme of side dry polder. While in many ways specific (e.g. not entailing local resistance), the case exemplifies recent shifts (and respective struggles) within flood risk management, including the increasing importance attributed to complex, catchment-wide perspectives, joint local and scientific knowledge, participatory decision-making processes, or implementation of nature-based and hybrid solutions.

## Introduction

Impacts of climate change are increasingly apparent; correspondingly, adaptation-related interventions become an imperative within policy-making processes, and a regular part of people’s everyday lives (Quinn et al. 2023). The procedural part of adaptation garners still more attention, including the key role assigned to transparent governance encouraging participatory processes and diverse approaches, but also reconciling varied technical, political, economic, environmental, or social interests (Potočki et al. 2022; Singh et al. 2022). Such issues and tasks come to the fore even more when considering extreme weather/climate events, amongst which floods belong to the most recurrent and disastrous ones (Hagos et al. 2022; Yin et al. 2022). Moreover, thanks to factors such as intensification of extreme precipitation events, pressure on river systems through socio-economic development, or inappropriate land-use changes (Collentine and Futter 2018; Raška et al. 2022), projections suggest that floods’ frequency, associated losses, and manifold cross-sectoral repercussions (Akukwe et al. 2023) will continue to increase (Hartmann et al. 2019; Ruangpan et al. 2021).

### Theoretical and conceptual frameworks

Recent flood risk management (FRM) echoes the respective challenges; gradually moving from the primarily technocratic approaches focused chiefly on flood defence, it aims to reflect more the need for holistic, integrative, inclusionary, and sustainable solutions to the manifold flood-related issues (Begg 2018; Collentine and Futter 2018). Understanding that flood risk cannot be completely removed (Geaves and Penning-Rowsell 2015), strategies striving to deflect the inundation have been superseded by mitigation- and adaptation-centred interventions (Walczykiewicz 2015; Albrecht and Hartmann 2021). Increasingly emphasized is the need to complement the structural measures (e.g. dams) with means such as spatial/land-use planning, emergency management, or early warnings (Begg 2018), and with efforts to bolster water retention within floodplains, (semi-) natural reservoirs, or wetlands, commonly subsumed under the umbrella of “nature-based solutions” (NBS) or hybrid solutions (such as dry polders), the latter ones combining both the technical/“grey” and nature-based/“green” elements (Collentine and Futter 2018; Hartmann et al. 2019; Raška et al. 2022).

A more “joined up” and holistic approach to water management throughout the hydrological cycle has been recognized as crucial (Collentine and Futter 2018) as well; accordingly, the issues of spatial fit, scale, and relations such as the upstream–downstream ones (Seher and Löschner 2018; Raška et al. 2022) are emphasized, including the need for an integrated, catchment-wide perspective (Collentine and Futter 2018; Hartmann et al. 2018). The interrelatedness of water management and land is highlighted as well; floods can be caused or exacerbated by (inappropriate) land use (Vári et al. 2022), while flood-protection/mitigation measures by themselves impact the ways the land is used (Walczykiewicz 2015; Albrecht and Hartmann 2021). Stronger integration of FRM with spatial and land-use planning is thus called for (Albrecht and Hartmann 2021; Slámová et al. 2021; Raška et al. 2022; Solín and Sládeková Madajová 2023).

Another hallmark of recent FRM developments is the idea of “shared responsibility” (Andráško 2021), meaning that a much wider array of actors/stakeholders than just governments, authorities/officials, and “experts” are now expected to be actively involved in, and accountable for flood-related activities (Begg 2018; Andráško 2021; Forsyth et al. 2023). This shift towards a more “societal” FRM (Begg et al. 2018) has been fostered through documents such as the Sendai Framework for Disaster Risk Reduction 2015–2030 (Cox et al. 2019), the European Union Water Framework Directive, or the European Floods Directive (Jager et al. 2016; Slavikova 2018). Aiming to employ varied resources across different levels of society (Geaves and Penning-Rowsell 2015), stakeholders’ expertise and a joint/“hybrid” knowledge (Reed 2008) of scientists/experts, policy-makers, local stakeholders, and communities are accentuated (Islam et al. 2018; Matczak and Hegger 2021) and assumed to aid the justification and robustness to the steps taken (Ruangpan et al. 2021; Potočki et al. 2022). The focus then moves to a transparent, inclusive, and participatory governance reflecting the varied everyday realities (Reed 2008; Quinn et al. 2023). These developments are not without issues though. The interest in participation is not always zealous, and the official declarations often simply do not line up with reality (Slavikova 2018; Andráško 2021). Another issue is that involving a range of stakeholders usually means involving also their more or less rival/conflicting views, preferences/priorities, and interests (Awakul and Ogunlana 2002; Pasquier et al. 2020; Zolghadr-Asli et al. 2021).

Drawing on the respective frameworks, concepts, and findings, in this study we answer the call for further research on planning, decision-making, stakeholder involvement, or implementation aspects of water-related projects (Hartmann et al. 2018; Turkelboom et al. 2021), but also for addressing these topics within the specific context of the post-socialist planning regimes (Raška et al. 2023). Thus, our case study focuses on the Skalička Waterwork (SWW), a largely debated, media-covered, and conflict-associated water-related project in the Czech Republic (CR). Based on several sources of data, we strived to trace back and reconstruct the evolution of the project, including the identification of its main tipping points. To specify the goals of our study, three main research questions were set:

- How did the project evolve, including the changing preferences for its particular variants?
- How was the project’s development, including the planning and decision-making processes, framed by the main stakeholders involved?
- Whether and (if so) how did the project’s development reflect recent shifts within the FRM and respective ways of governance?

The remainder of the paper is organized as follows: first, we introduce the context of the case study, including the information on floods and FRM developments in the CR and selected water-related projects with conflicting natures, followed by a factual description of the case study and SWW evolution (Section “Background”). After reporting on the materials used and methodical procedures applied (Section “Materials and methods”), we then proceed with the results of the study (Section “Results”), discussion of our findings (Section “Discussion”), and concluding remarks (Section “Conclusion”).

## BACKGROUND

### Floods and FRM in the Czech Republic

Czech history is rich in floods, the country’s most important (and destructive) weather extreme events (Brázdil et al. 2006; Dolák et al. 2013). Accordingly, various protection/mitigation measures were adopted; chiefly as regards the twentieth century, those technical/structural ones prevailed though (Gacko et al. 2020), including the construction of nearly 600 water reservoirs, around 120 out of which were large dams (Bera and Daněk 2018). Manifold land-use changes such as merging plots of arable land, changing mixed forests into coniferous monocultures, modifications of riverbeds, or shortening of the river courses took place as well, largely decreasing the potential for natural water retention (Vaishar et al. 2000; Brázdil et al. 2011).

The second half of the twentieth century, described as the period of flood tranquillity/silence (Klemešová 2016; Slavikova 2018), ended with two major/extreme floods, namely the 1997 event, affecting the north-eastern part of the country (Klemešová and Andráško 2015; Duží et al. 2017), and the 2002 flood, hitting the southwestern part of the CR, yet later also other regions (Slavikova 2018). A series of minor to major floods (e.g. in 2006, 2009, 2010, and 2013) followed (Vávra et al. 2017; Bera and Daněk 2018), altogether stimulating the developments of the Czech FRM. Incentivized also by the country’s administrative decentralization and adoption of the European Water Framework Directive or the Flood Directive, several strategic and legislative documents (such as the Czech Flood-Protection Strategy) were approved (Duží et al. 2017; Slavikova 2018), highlighting the need of flood prevention and risk and responsibility sharing (Andráško et al. 2020).

Despite these developments though, the FRM in CR is still largely hierarchical, with the key role in decision-making assigned to the central government, and participation of a wider spectrum of actors lagging what was proclaimed (Vávra et al. 2017; Bera and Daněk 2018); also, while the nature-based and hybrid solutions increasingly find their way into the Czech FRM (PMO 2023), the reliance on technical/structural measures, designed by engineering experts of state river basin administrators (Slavikova 2018), continues to prevail (Klemešová and Andráško 2015).

### Examples of water-related projects and conflicts in the CR

Considering the number of dams/water reservoirs built (Section “Floods and FRM in the Czech Republic”), it is no surprise that these projects were often controversial and met with protests. The sociopolitical changes after 1989 caused the number of planned projects to be reduced, and environmental and socio-economic factors were increasingly taken into account. Current plans still consider building water dams, the process of planning and construction is often delayed though, partly also because of the continual empowering of civic society and more emphasis placed on public discussions and interests of stakeholders such as municipalities, NGOs, or landowners. Table 1 summarizes some of the key projects, often stretching from a relatively distant past to the present. Standing out is the river canal connecting three major Central European rivers: the Danube, Odra, and Elbe (D-O-L); discussed since the 1930s, and with reinvigorated political interests during the 1990s, it became largely disputed throughout society. The SWW was intended to become a part of this project.

**Table 1:** Examples of planned or implemented water-related projects in the CR

Name	Purpose	Time frame	Current state	Disputes, conflicting views
D-O-L water corridor	Mainly transport	1930s–2023	Partial realizations in Danube River Reconsiderations in governmental strategic documents over time, and finally cancellation of the project	Strong protests from environmental movements and selected municipalities, different opinions amongst experts/scholars
Nové Mlýny water dam	Multipurpose	1970s–1989	Constructed, consisting of three connected water dams	Protests from environmental movements, and rescue actions (water plants and animals); disputes also after construction was finished
Slezská Harta water dam	Multipurpose	1987–1997	Constructed	Low response from civic society. Nearly no protests
Dlouhé Stráně water dam	Energy production	1978–1996	Constructed. Two dams, upper and bottom, ensure electricity via the drawing process	Some protests from environmental movement (location in PLA Jeseníky)
Nové Heřmínovy water dam	Multipurpose	1953-present	Phase of project implementation. The government approved the realization of the water dam project in 2008. Nearly all plots have been bought up by the state	Environmental movements and selected municipalities still protesting. Their alternative solution was not approved
Water work Skalička	Changing concepts	1954- present	Changes and deviations from the original project. Finally, the government approved the project of the dry side polder variant (2022). Two-thirds of plots are bought up by the state	The environmental movement and experts/academia protested and proposed an alternative solution. Wide public discussion and media attention
Žichlínek polder	Flood protection	2002–2008	Constructed, accompanied by the revitalization of the river. All plots were bought up by the state	In general project is accepted, accompanied by the revitalization of the river—positive perceptions
Vlachovice water dam	Multiple	1954-present	Phase of project implementation, territorial permission is given. The government approved the realization of the dam (2018), accompanied by a nature-based solution	Supported by local municipalities, incorporated into the decision-making process, and high-level communication in all stages of the project

Multipurpose usually means a combination of protection/mitigation goals, energy production, recreation, and irrigation functions. *Source*: Elaborated by the authors based on publicly available sources

### The SWW case

**Fig. 1:**
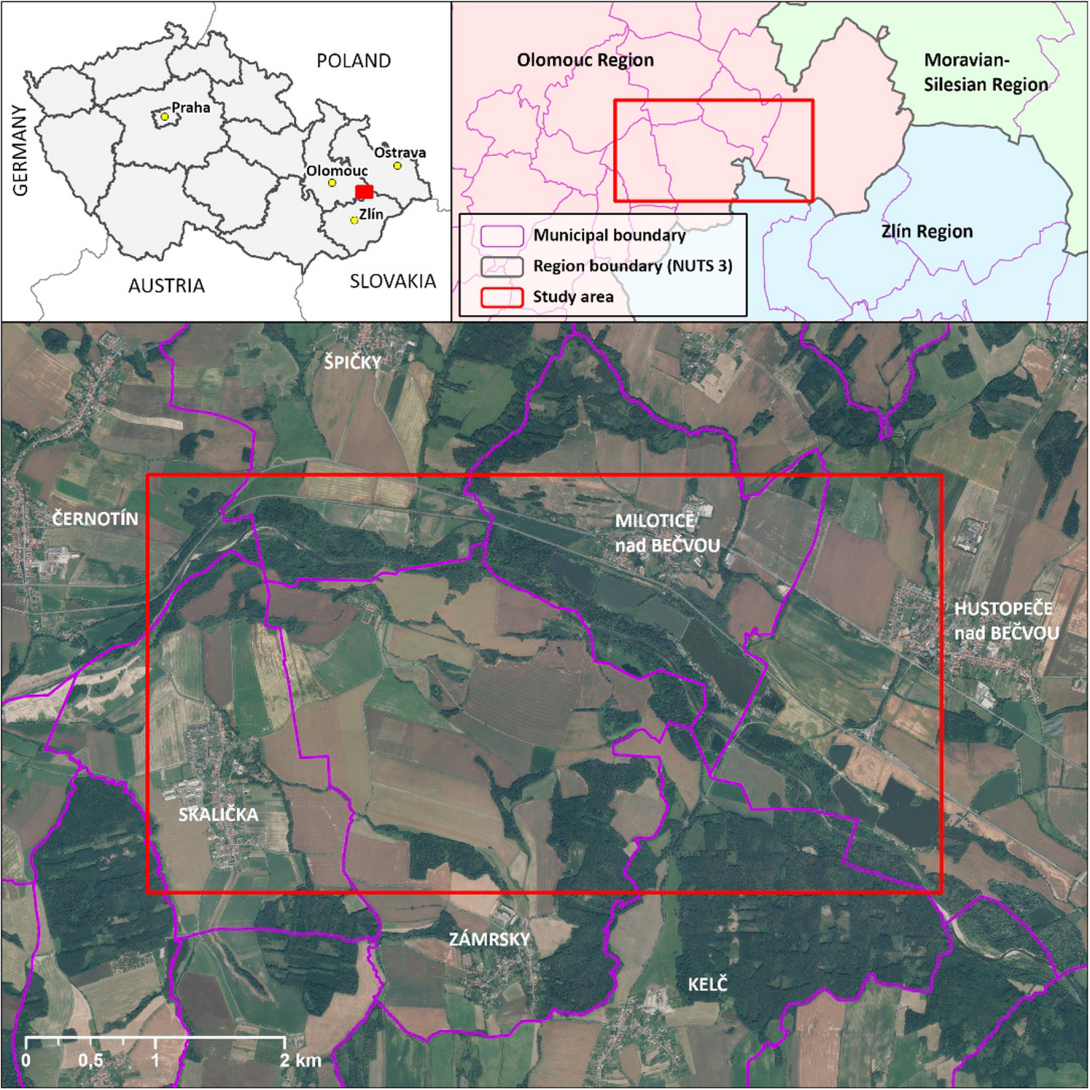
Case study area. Source of Map: Data50, 2022 © Český úřad zeměměřický a katastrální, www.cuzk.cz>, ©ArcČR, ARCDATA PRAHA, ZÚ, ČSÚ, 2016

The Skalička case study is located in the north-eastern part of the CR on Bečva (Fig. 1), a 61.6 km long river with a highly fluctuating flow rate (Duží et al. 2017). Some technical solutions were implemented within its basin in the past, but it has never been crossed/dammed by any large-scale measure (Čermák 2010; Krejčí 2016). The disastrous 1997 floods and several later events (e.g. in 2006, 2009, and 2010) recently affected the area; in addition, local properties are repeatedly flooded from nearby streams as a result of intense precipitation and soil saturation (Duží et al. 2017).

**Fig. 2:**
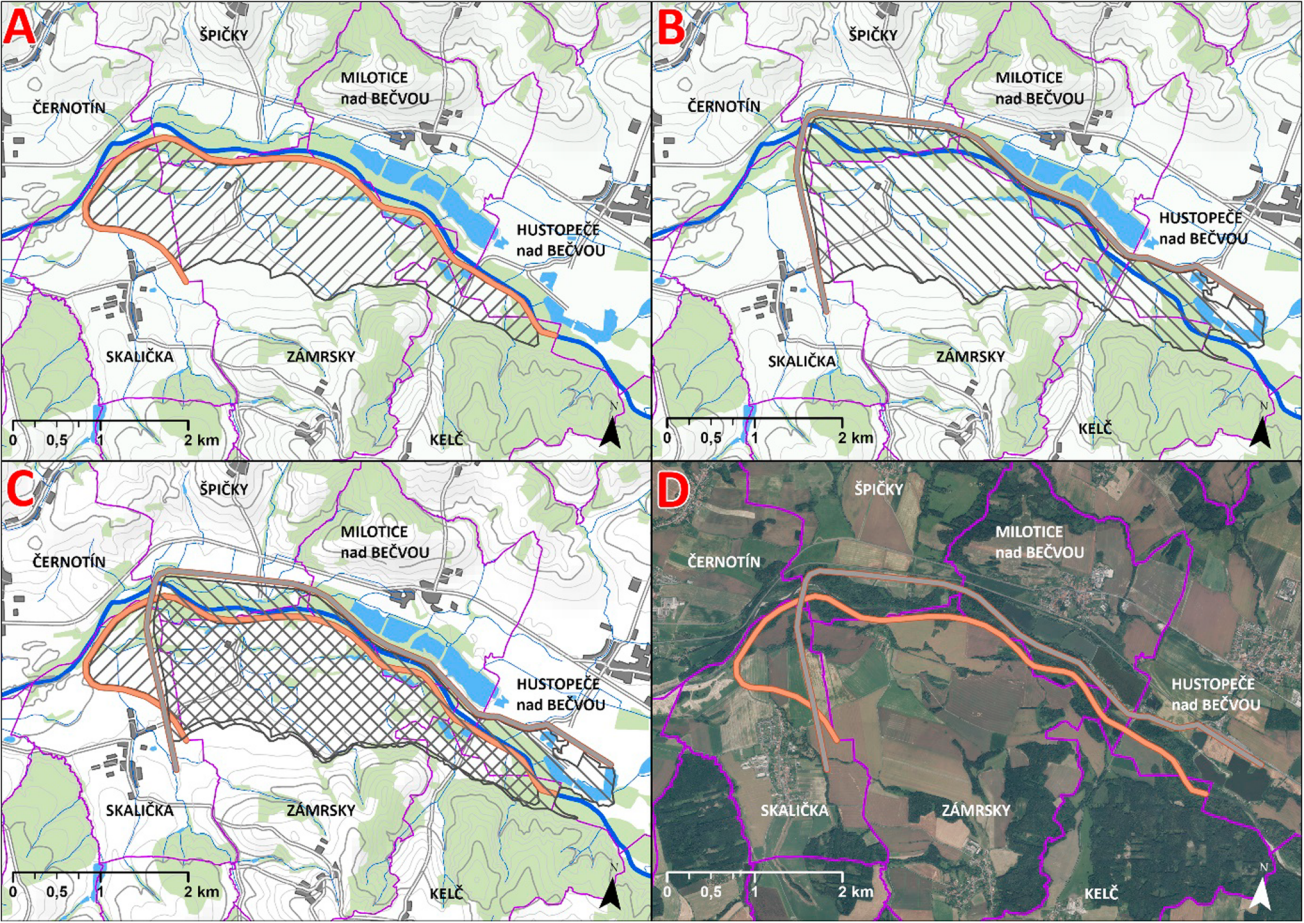
Visualization of the SWW two most debated variants (A: side dry polder, B: on-flow water dam, C: A and B variant overlapped, D: A and B variant on an aerial photo. Source of Map: Data50, 2022 © Český úřad zeměměřický a katastrální, www.cuzk.cz>, ©ArcČR, ARCDATA PRAHA, ZÚ, ČSÚ, 2016

Since 2011, the state Morava River Basin Administration (PMO) elaborated proposals for measures along the Bečva River to provide flood protection. The first phase of the project focused on local measures, and although technical solutions have been prioritized, several parts of the river were, also thanks to the activities of local NGOs, left for natural revitalization. (Some of these were approved as protected areas.) The second phase then concerned the construction of a large-scale measure/waterwork, an idea that originally dates back to the 1950s and the intentions to construct a dam supplying water to the planned (yet never realized) Blahutovice nuclear power plant. Large parts of the land were set aside accordingly; nevertheless, the project was later put at bay. The flood in 1997 renewed interest in it (Geršl and Konečný 2018; PMO 2023), yet the vision was changed to a large on-flow polder Teplice. As a proclaimed response to the episodes of droughts, the project was reconsidered once again for a multipurpose, approximately 6.5 km^2^ large on-flow dam/water reservoir, partly relocated, and renamed to SWW in 2015. The process of buying up land was approved by the government and started in 2016. The same year, the civic association Union for the River Morava introduced an alternative variant of a side/lateral dry polder not directly affecting the flow of the Bečva River; associated with other NGOs, academics/scholars, state administration representatives, or politicians within the joint initiative called United Bečva, since 2017 they actively entered the decision-making process. Several threats the on-flow dam can pose for the natural flow of the river, local hydrogeology, or biodiversity were articulated, which, supposedly, the side polder variant should diminish. The initiative not only drew additional media and public attention to the case, later even bolstered by the Bečva River poisoning through leakage of chemicals in 2020, but also managed to initiate further analyses to be conducted. Their proposal was incorporated into the expert-based, multicriteria analysis (MCA) of the project, arranged in 2021 by the PMO and the Ministry of Agriculture; a set of variants were assessed (Fig. 2), including the on-flow and side ones of both the dam and the polder, and, for the sake of comparison, also a null variant.

**Fig. 3:**
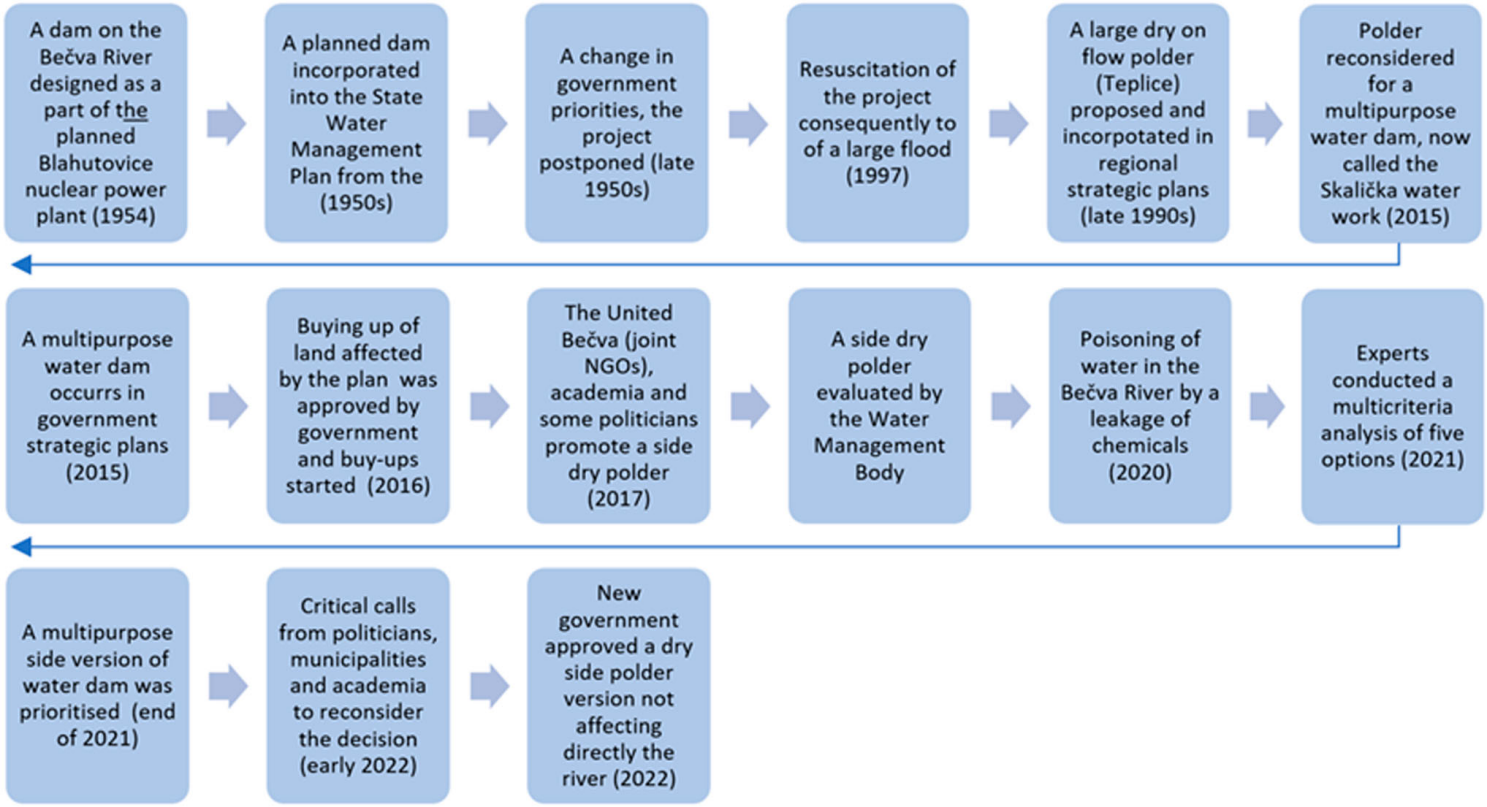
Key phases of the SWW. Source: elaborated by authors

The side dam variant went out of the analysis as the best option (PMO 2023), yet, once again, met with continuing protests. Finally, in 2022, the newly mandated Ministry of Agriculture and the new government approved the side polder version (see Fig. 3 for the key phases of the project’s development).

## Materials and methods

**Fig. 4:**
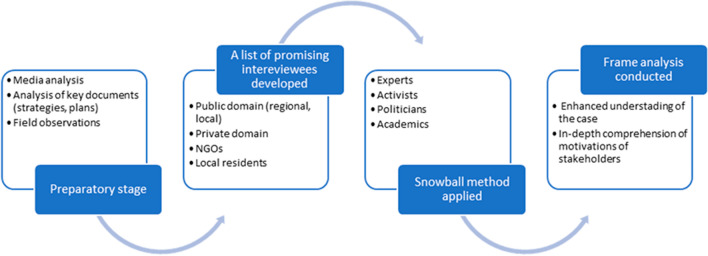
Phases of the research. Source: elaborated by authors

Following the methodical recommendations of Raška et al. (2023), we traced back, explored, and retrospectively reconstructed the evolution of the SWW project (Fig. 4).

The interviews with stakeholders, defined as those persons who have a stake or special interest in the case (Welp et al. 2006), were the crucial source of our information. The interviewees were selected based on the initial analysis of the case (identification of the main parties involved) and later added through the snowball method; the sample included stakeholders from governmental institutions/state administration and citizen initiatives/NGOs, as well as politicians, members of Local Action Groups (LAG), and researchers (Table 2).

**Table 2:** List of the interviewees

Category	Gender	Position and reasons for interview	Code
Local government	F	Local government representative of a potentially highly affected municipality	LG
NGO	M	Local NGO representative, engaged in nature protection and environmental education	NGO1
Education sector	M	Teacher, regional museum employee, engaged in nature protection and environmental education	EDU
State administration	F	Representative of karst cave administration, active in research and raising public awareness about karst uniqueness	SA1
NGO	M	LAG representative, engaged in regional socio-economic development	NGO2
NGO	M	LAG representative, engaged in regional socio-economic development	NGO3
NGO	F	LAG representative, engaged in regional socio-economic development	NGO4
Academia	M	Researcher focused on socio-economic development in rural areas	ACAD1
Politician	F	Senator engaged in the affected region’s development and political solution of the case	PO
NGO	M	Representative of the NGO engaged in the proposal of an alternative variant	NGO5
State administration	M	Representative of the state river basin administration	SA2
State administration	M	Representative of the state river basin administration	SA3
Academia	M	Researcher focused on hydrogeology, observer, cooperation with NGOs	ACAD2

Focused on four main topics, each covered by a set of questions (Table 3), the semi-structured interviews were conducted in 2021 and 2022.

**Table 3:** Thematic focus and questions used in the interviews

Thematic focus	Questions
Information on the project and its development	(a) How do you perceive, essentially, the situation regarding the SWW project?
	(b) What is the current state of the project?
Prioritization of a particular variant	(c) How do you perceive the reasons for the project’s variants prioritization?
	(d) Do you see these reasons as adequate and relevant?
	(e) Are there any (further) alternative solutions to the project?
The roles of actors and the nature of the conflict	(f) How do you see your (and other actors’) roles in the process?
	(g) Has the situation a conflicting nature?
The future of the project	(h) What were (or will be) the key steps to the project’s realization?
	(i) Which barriers (if any) do you perceive as crucial for the project’s realization?

All interviewees were informed about the goals of the study and how the information acquired will be processed and utilized. Having their consent, each interview was recorded. Transcriptions of the interviews were investigated through the frame analysis (Singh et al. 2022) to identify and highlight the stakeholders’ perceptions of the case (Pasquier et al. 2020).

A deeper understanding of the case was acquired through data from an online survey (2021–2022) centred on the SWW-related perceptions of the inhabitants of the municipalities directly affected by the project (Fig. 1). Respondents (N = 103) were informed of the goals of the study and how the data would be utilized, and their participation was voluntary and based on prior consent. The sample covered approximately 3% of the local population aged 15+ (2 to 5% in each of the municipalities), yet in terms of demographic characteristics, it was not completely representative. Therefore, we focused only on selected aggregate statistics complementing the interviews-based findings.

## Results

### Origins of the conflict

All the interviewees agreed that the disastrous floods in 1997 triggered the resurrection of the idea of building a large-scale flood-protection measure on the river Bečva, and thus, this event represents the first tipping point in the recent record of the case. The back then valid notion of the on-flow dry polder Teplice was, however, not without problems, since although it was planned, *“nobody knew where to start” (PO)*.

Another 8 years later, mainly due to recognition of the need to deal also with droughts (*PO: “the big drought came”*), the project was *“reconsidered … to serve also as a water reservoir” (SA2)*, specified as a multifunctional dam with a constant water level (“the wet variant”). Other drivers behind the development were, however, implied as well, such as the plans for the D-O-L canal (*NGO3: “[the dam] was supposed to be a part of a magnificent construction”*; *PO: “the push to build the D-O-L began”*). Regardless, the “polder to dam” shift was the second tipping point of the case and the moment when *“the problem appeared” (NGO1)*.

In reaction, two main currents of opposition emerged. For the first one, *“the source of concern seemed to be the idea of crossing/damming the profile of the river basin” (NGO1)*, which would mean complex repercussions for the local environment; the Union for the Morava River thus proposed the alternative of the side dry polder (Krejčí 2016), which would leave the river basin intact. The second current of resistance pointed to the *“permanent hydrostatic loading endangering the stability of the spa waters” (SA1)*, having potentially adverse consequences for the nearby Teplice spa and Hranice Karst. *“That was the moment when we, as experts, raised our finger” (SA1)*. It was argued that *“the PMO wanted to choose that option without a hydrogeological assessment” (SA1)*.

Both currents succeeded. Not only did they enforce *“the [hydrogeological] survey [to be conducted]” (PO)*, but the side polder variant was included in further consideration as well.

### Dispute over the “right” solution

The representatives of the state river basin administration (the PMO) saw the main problem in the very word “dam”, which, because of the heritage of the communist era, still *“has … in the CR … negative connotations” (SA2)*. Thus *“once the word appeared … clashes with some other organizations began” (SA3)*.

The debate about the “right” solution was much more complex though. After 1997, smaller-scale measures began to be implemented within the affected municipalities *(NGO2: “The mayors understood that it is necessary to make even small interventions”*). Technical interventions (e.g. embankment dikes) appeared, which were, however, only *“increasing peak flow … making the downstream spillage even worse” (PO)*. Also, the NBS were increasingly implemented (*NGO2: “kudos to the municipalities doing that”*) and showed up to be effective chiefly in terms of managing the flash floods (*NGO3: “the results are tangible”*). Some NBS have been realized in the catchment of Bečva based on the initiatives of both local NGOs and the PMO (*NGO1: “We made a lot of effort to establish the Doubrava-Bečva reservation”*).

The importance of the NBS was commonly recognized, yet the views about their effectiveness differed. Thus, there were views that local NBS or hybrid measures would suffice the water management needs in the region (*SA1: “Based on common sense, I'd say it [the large-scale measure] would not happen and we’ll work towards a system of [smaller] dry polders”; NGO5: “it can be solved without any large-scale measure”, EDU: “it is better to retain water … in several places than to concentrate it in one”*). The representatives of the PMO were also in favour of the NBS (*SA3: “as many of them as possible should be implemented”*) yet pointed out their complementary function (*SA2: “both [kinds of measures] are needed”; SA3: “a wetland cannot be an alternative to a water reservoir if talking about supplying people with drinking water”*).

Regardless of these debates, the development of the SWW project crystallized into the dispute between the “wet variant”/the dam proponents (mainly PMO), and its opponents, now joined under the initiative United Bečva, pushing for the variant of side dry polder.

### Trying to differentiate the facts and opinions

The lack of belief in the opponent’s expertise largely accompanied the dispute (*SA1: “The PMO has had some studies painted, how accurate they are, I'd rather not comment on that…”*). One of the main objections against the dam was that the respective plans and documentation did not consider enough the specifics and complexity of the territory (*SA1: “[because of] the geological structure … building any kind of dam here is nonsense”; PO: “The documentation … was based on insufficient surveys”*). Furthermore, it has been indicated that only thanks to the suggestion of the side polder alternative with *“a high professional level” (NGO5)*, the PMO realized there are also other options available (*ACAD2: “It's like me doing your work instead of you”*). The PMO representatives did not share these views, saying that consideration of other options took place only because of the pressure of the *“ecological organizations” (SA2)*, and the alternative scheme needed to be further developed *“to be feasible and functional” (SA3)*.

In any case, the PMO initialized the MCA (the third tipping point); involving several variants of the SWW, relying on a range of experts/stakeholders, and employing a *“very scrupulous”* approach, it aimed to provide an *“independent/objective and complex assessment”*, identify the best solution, and *“avoid disputes and blaming” (SA2)*. It did not work though, and *“we [PMO] were accused that it was just some kind of contract, to ensure it will come out in a certain desired way” (SA2)*. Indeed, objections emerged, suggesting that the MCA was *“done in a hurry” (SA1)*, methodically outdated *(ACAD2)*, not sufficiently *“balanced and transparent” (SA1)*, and *“did not employ proper weights [of the criteria] (PO)”*.

### Participation and communication

The communication between the actors was not easygoing. The PMO representatives stated that their opposition often relied on *“untrue” (SA2)* and *“taken out of the context” (SA3)* claims. *“Against this … is very difficult to communicate … I doubt anyone even read the analysis properly” (SA3)*. The other party, on the other hand, pointed out that *“When we tried to present … arguments … they [PMO] belittled it and did not take it seriously” (SA1)*. These issues translated also into communication with locals. The PMO claimed that they strived for *“maximum dialogue” (SA2)*, communicating openly through public meetings/presentations; the opposition, however, suggested that the information provided was biased *(SA1: “[P]eople were not informed well”; NGO1: “no one told them [the locals and mayors] … about the negatives … so there would be no resistance against the dam”)*, and those presenting things otherwise, were excluded *(SA1: “they [PMO] didn't invite us to it [meetings with mayors], even though we tried [to get there]”)*. The opposition then spreads also information about the risks *(EDU: “[W]e created a leaflet/comic that explains it to people”)*, disavowing the supposedly unrealistic/unattainable promises, including the largely debated recreational function (*ACAD2: “they manipulated with those stakeholders … that there will be recreation”; SA1: “boats on the water are a romantic idea …during droughts, there won't be romance, but mud and lots of mosquitoes”)*. The locally based actors corroborated these issues *(NGO2: “pleasant arguments regarding the recreation … I don’t really believe it”; LG: “At that time [2016] … promised us the moon … an important touristic place … [after getting more information] we [the mayors] began to think this would not be a suitable option”)*, including the lack of proper information *(NGO2: “On the part of the PMO, information provision is quite poor … the visualization of the dam gives it the kiss of death … [it] is half-baked and rather scares and discourages people”; LG: “we didn’t get the complete information as we should”)*.

Notions of pressures and imbalance of power and resources appeared as well. Polder proponents suggested they had *“fewer options than dam promoters” (EDU)*, the negotiations were *“an unequal battle”*, and because of *“the strain and psychological demands” (ACAD2)* the process was *“sad and exhausting” (SA1)*. The other party complained as well, pointing to the undue media coverage and *“politicization”* of the case *“to push them to approve the [polder] proposal” (SA2).* This “push” was admitted by the dam’s opposition, yet it was interpreted as necessary to confront *“that Goliath [which] does not work well” (ACAD2)*.

### Standpoints of the communities

Based on the interviews, the standpoints of local representatives towards the project changed from initial support of the dam—mostly because of its (promised) recreational function—to *“confusion”* and *“disillusionment” (NGO1, EDU)* once informed also about the negatives. Nevertheless, the municipalities employed varied strategies to get the best for them *(SA1: “Some municipalities … sold their municipal land [especially those more distant] … some do not want to sell their land, they hold it and have serious objections”)*. Concerns about having the construction close to their built-up/residential areas promoted some municipalities’ higher engagement in the decision-making process *(LG: “[P]eople would have the dike right next to their gardens/houses … we want to influence it somehow”)*. Understanding that achieving project cancellation is unattainable *(LG: “[W]e wrote piles of letters … it is impossible”)*, they joined the dam opposition to amplify their voice in the decision-making process *(LG: “we will look for some … reconciliation”)*. The unpleasant experience with lack of proper communication *(LG: “The PMO didn't care at all”)* was then seen as one of the reasons why *“the mayors … do not tend to get more involved” (EDU)*. Other interviewees (e.g. NGO2 to 4) also suggested that the local representatives simply have a multitude of other, much more current/actual issues to solve, while the SWW is something too distant, both in terms of time and priorities.

Local inhabitants were said by our interviewees to be *“mostly uninterested”* (NGO1) in the case. Some reasons why *“very few locals would deal with it” (EDU)* were that the whole dispute was distant and obscure for them (NGO2 to 4), and they lacked enough expertise and relevant information (ACAD2). Fatigue of the long development of the project was mentioned as well *(EDU: “It's taking an awfully long time”)*, yet also the lack of belief that *“someone will take their opinions into account” (EDU)*.

Another aspect is that the SWW *“will have a minimal [direct] impact on the local population” (NGO2)*, and with only a few exceptions, it will not involve relocations. The buyouts of the land went smoothly *(LG: “[P]eople are selling”)*, also because the area is under construction closure and within flood zones *(SA1: “they didn’t know what to do with it [the land] anyway”)*; the buyouts then are *“a welcomed opportunity … to valorize these lands” (SA1)* and make *“nice money” (LG)*. In terms of the upstream–downstream relations, it was stated that *“the locals do not need it [the large-scale measure]” (NGO1)*, but *“it is necessary to protect the residents downstream, and we … should be solidary” (LG)*.

**Fig. 5:**
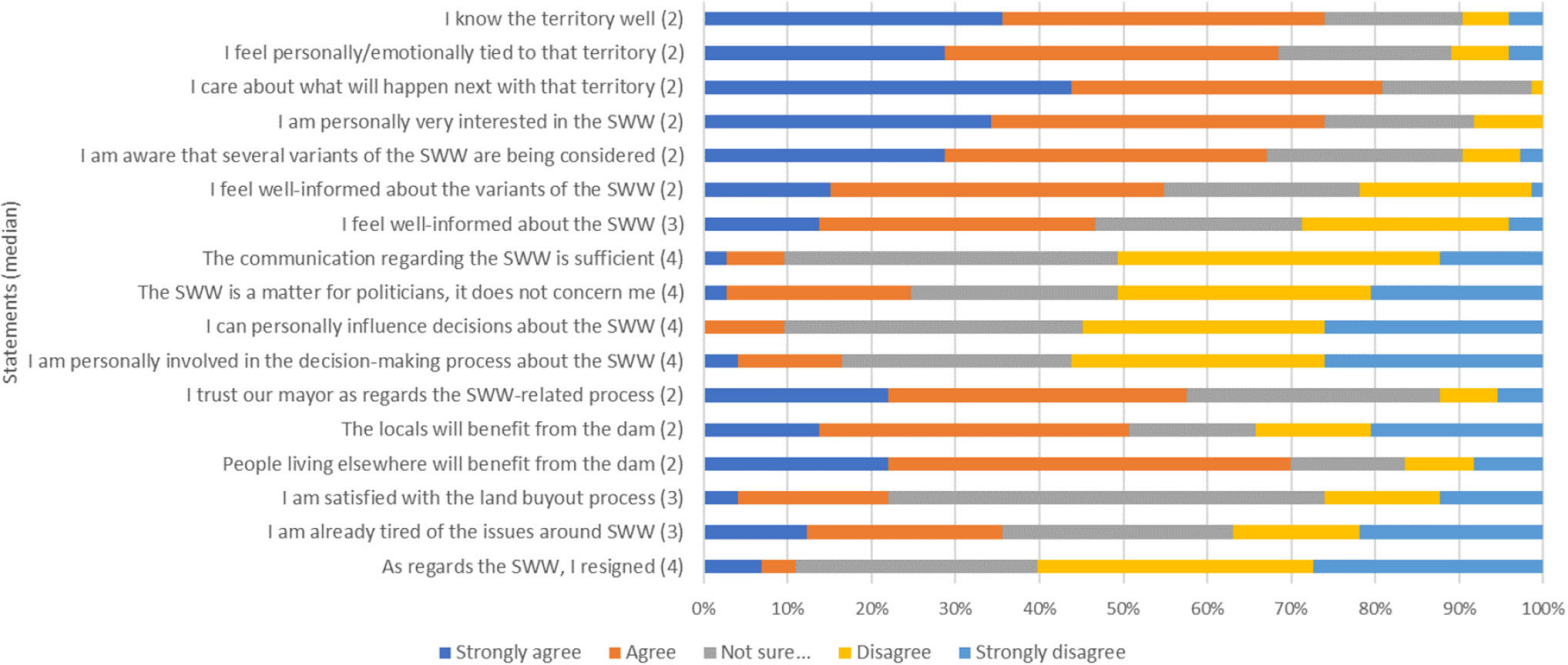
Respondents’ statements regarding the SWW project

The questionnaire survey results largely, yet not completely corroborated these views. Approximately one-third (30%) of the respondents stated they never heard of the project. As for the rest (Fig. 5), most of the respondents knew the affected territory well, even having an emotional tie to it, and caring for its future development. However, while mostly claiming they are “very interested” in the project, they also did not tend to get personally involved, relegating the responsibilities to their representatives. In more than half of the cases, the respondents also thought that the communication (from the PMO, government, etc.) regarding the SWW was insufficient and that they could not influence the respective decisions in any way. The project was perceived as beneficial both for locals and people living elsewhere/downstream. The notions of “resignation” or “fatigue” out of the project were not supported by our data.

**Fig. 6:**
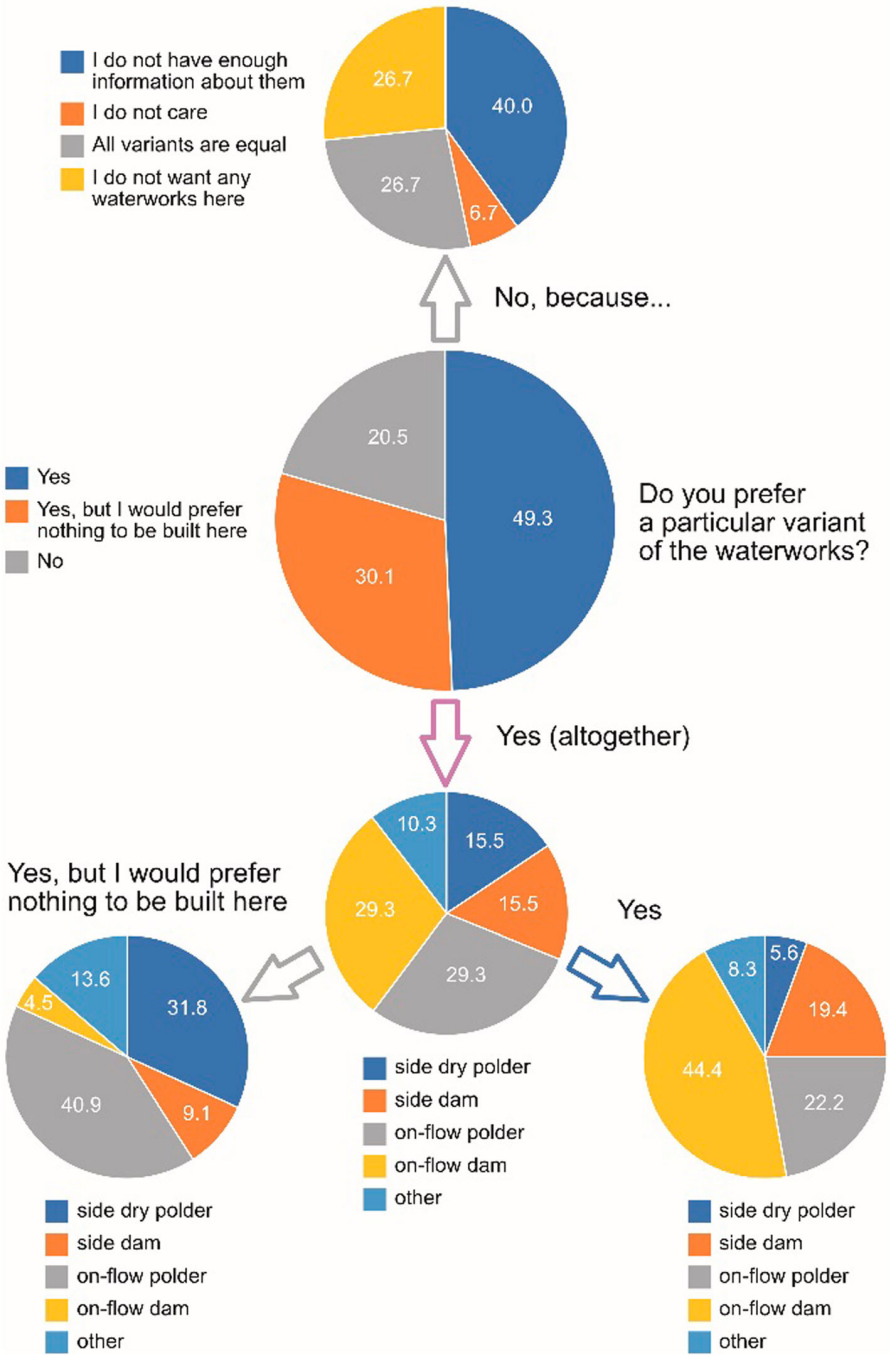
Respondents’ preferences for individual variants of the SWW

Respondents felt to be relatively well informed about variants of the SWW. Around 20% did not prefer any particular variant, mostly because of insufficient information (Fig. 6). If preferring any variant, the shares of those favouring the dam or the polder were relatively balanced; however, supporters of the polder were mostly those locals, who would prefer if nothing was built in the area at all (altogether, such a view was held by around 36% of the respondents).

### The final decision

The final tipping point of the case so far was eventually the government resolution and approval of the side dry polder as the variant to be built. Since the MCA identified the side dam as the best solution, the PMO representatives felt disappointed and *“demotivated”* by this *“political decision”*.

The dam opposition highlighted their success as a result of joint action (*PO: “Scientists and activists/environmentalist united”; ACAD2: “NGOs and professional organizations … scientists … linked together, … which played the crucial role”*); striving to *“deliver the protest as professionals” (SA1)*, they relied on *“truly strong arguments” (NGO1)*, including the potential destruction of the spa and proposal of alternative variant, seeing their activities as a proof *“that we have a functioning civil society here” (PO)*. Some members of the polder proponents group also noted that to succeed though, their protest “*had to reach the political circles as well” (NGO1)*, or that the local representatives perceived some steps of its “expert part”, at times, as lofty. Also, while the dam opposition succeeded, talking about a “victory” would be an exaggeration; for most of them, any large-scale measure was unnecessary in principle (*LG: “If it were up to me, I would wish there was nothing here”*), making even the polder variant *“a mega intervention to the landscape” (NGO5)*, a *“compromise” (e.g. SA1)* which had to be done to succeed at all.

### A conflict or a negotiation?

While acknowledging that *“at times, it was very intense” (LG)*, most participants agreed that the SWW-related process was more about *“complex negotiations” (PO)* than a conflict in the very sense of the word *(ACAD1: “A conflict? I didn't see it that way”)*.

Furthermore, although accusations of incompetence or bitter notions of manipulative techniques appeared regularly, the interviewees talked about each other with respect, and without unnecessary antipathy (*NGO1: “[W]e do not have an outright hostile relationship”*). Condescension was present as well; for the PMO representatives, their opposition replaced insufficient expertise with enthusiasm and pressure through media and politicians; for the locally based representatives, the case was an “expert versus expert” contention, by which they stood a bit aside; for the dam opposition, the river basin administrators were those *“influenced by the past” (EDU)*, a community of *“relatively high closedness” (ACAD2)*, still relying mostly on *“flood protection, which will solve nothing” (PO).* Nevertheless, it was also pointed out that the PMO representatives are parts of a system working towards *“a political assignment … [which] they had to implement” (SA1)*, taking their steps under *“enormous political pressure” (PO, ACAD2)* of the *“people who make decisions” (EDU)*, and, eventually, affect the course of action (*LG: “out of the blue … they [PMO] called us … the water reservoirs will be not supported anymore … well, a new political situation”*). The notions of some “hidden” or latent forces standing behind the processes of decision-making emerged within the interviews regularly (*LG: “[E]ven the PMO did not have the leverage to defend the chosen option … I don't know what exactly played a role in it”; ACAD2: “all these things have a background that we probably don't even know”*). Thus, while the protest against the dam was environmentally motivated, the participants were aware of its political and other connotations *(NGO3: “[I]t was … a political game”; EDU: “[it depends on the] political constellation of our country … [and] pressure from the concrete lobby and companies”; SA1: “The political line, where decisions are made”*).

### Future of the project

With the final decision, for most of those involved things are far from being done. Municipalities with residential areas in the vicinity of the dike will continue to *“fight for it to be placed further” (LG)*. The PMO will follow with *“further studies and surveys … [to] determine the parameters of the polder in more detail” (SA2).* The NGOs intend to continue *“promoting the naturalization of the Bečva River”* and further engage in the *“future character of the polder”* (NGO5). For those concerned about flood protection, the *“postponed again”* implementation of the measures raises concerns such as *“what if the big water comes [in the meantime] again…” (PO)*. Especially the locally based interviewees were then *“skeptical about the time horizon” (EDU)* of the SWW completion, projecting it to a distant/indefinite future (*NGO3: “[M]aybe in 10 or more years it will start … [but at that time] we may be discussing something completely different than SWW”*).

## Discussion

Protests against water-related projects are nothing exceptional (Atzl 2014; da Costa 2014; Wilmsen and Webber 2017), even if considering solely the context of the CEE countries (Harper 2005; Raška et al. 2023); nevertheless, it still holds the case of SWW is largely specific, yet at the same time also emblematic of recent developments (not only) within the Czech FRM (Table 4).

**Table 4:** Specific and emblematic features of the SWW case/project

Specific	Emblematic
No large-scale relocations, no flooding of residential areas	The multitude of stakeholders/participants involved
No land-related issues	Conflicting views and interests
No protests of the local inhabitants	The issues of redefining the state’s and other actors’ roles
Extraordinary involvement of scientists/experts on both sides of the conflict	Complex, catchment-wide perspective applied
Alternative variant proposal	Environmental and flood-protection concerns, yet with a political background
Locals “excluded” by both main parties	From flood protection/defence to flood mitigation
The success of the opposition	From structural measures to hybrid/NBS solutions
Economic aspect not prioritized	Local and scientific knowledge joined
Long-term development/history of the project, several twists, and tipping points	Importance of adaptation to/mitigation of weather extremes recognized

### Development of the project

Reflecting the constant change of societal values and expectations affecting the views of “optimal” solutions (Raška et al. 2023), several twists and tipping points accompanied the lengthy development of the project, eventually described as *“strange: first a dry polder, then a dam, then … a lateral dry polder, then … a wet lateral variant … and eventually, we have the dry variant” (LG)*.

The involvement of several stakeholders played a prime role in the recent phase of the case, conducing to a situation in which the participants may acknowledge common goals (such as the weather extremes mitigation), yet differ in views of how to reach them (Zolghadr-Asli et al. 2021). In line with recent findings (Collentine and Futter 2018; Hartmann et al. 2019), the dam proponents claimed that small-scale measures can complement, yet not replace a major solution, while their opponents, although in some cases not contradicting the view principally, insisted that it does not suit the specifics of the area in question, especially if the major solution would be a water reservoir. Turning the case into the “dam versus polder” dispute, they thus came up with the proposal of an alternative variant, a unique strategy adopted in a situation when the abandonment of the project was unattainable; the idea of a side dry polder found its support in the up-to-date findings and FRM best practices, demonstrating the polders’ large retaining capacities and ability to reduce flood peaks while allowing for further agricultural use (Albrecht and Hartmann 2021). The option was also seen as a means of preventing numerous negative repercussions for the local landscape (Geršl and Konečný 2018) which constituted, eventually, the key point of concern of the wet variant’s opposition. Repeatedly referring to Bečva as “a gravel-bearing river”, detrimental effects of the on-flow project construction on the run-off regime, transport of materials, or local biodiversity, altogether affecting the floodplains’ ecosystem functions (Jakubínský et al. 2021; Vári et al. 2022), have been highlighted. Consistent with the crucial role attributed to catchment-wide perspectives (Hartmann et al. 2018; Matczak and Hegger 2021; Máčka et al. 2022), the emphasis placed on the complex landscape components’ relations led to the probably decisive “against the dam” argument, namely the endangerment of the ground/mineral waters and local spa and karst. Interestingly, the economic aspect was much less emphasized, although currently considered one of the key elements of the relevant decision-making/planning processes (Turkelboom et al. 2021; Raška et al. 2023). The MCA concluded that the dam variants are more economically efficient than the polder, in part also because of revenues from the electricity production and drinking water supply. Nevertheless, dam opponents believed that the polder was economically more advantageous, so *“even if it would be more expensive, the costs of the dam’s consequences would exceed it” (NGO1)*. While specifics of each case always need to be taken into account (Ungvári and Kis 2022), findings from elsewhere indeed imply that retention-centred solutions are more cost-effective due to higher co-benefits for people and biodiversity, and lower maintenance costs and chances of catastrophic failure (Turkelboom et al. 2021; Vári et al. 2022); the view is underlined if—as in the SWW case—the housing and agriculture opportunity costs are low (Turkelboom et al. 2021), and the land is available (Hartmann et al. 2019).

### Roles of actors

Thanks to their ability to bear the role of a “watchdog” (Awakul and Ogunlana 2002), in the SWW case, like elsewhere (Schulz and Adams 2022), as crucial proved to be the performance of the NGOs; their activities decidedly affected the key phase of the planning/decision-making process, including the assessment of alternative variants of the project. Building upon contextual features such as the pre-existing action groups (Geaves and Penning-Rowsell 2015), they were able to overcome common barriers such as bureaucracy or accusations of lacking expertise (Potočki et al. 2022) through the united commitment of diverse stakeholders; the integration of local and scientific knowledge not only allowed for a comprehensive understanding of the complex local systems and evaluating the appropriateness of the project (Reed 2008) but, eventually, provided for the key argumentation within the case.

The role of the river basin administrators was troublesome; on the one hand, they were accused of “traditional” reliance on the structural measures and expectations that the public will support, yet not so much actively participate in, water-resources projects planned by “professionals”. The rigidity of this approach was, supposedly, the reason why instead of pushing for the project’s complete dismissal, the dam’s opposition rather aimed to propose a more river-, landscape-, and nature-friendly alternative. On the other hand, the situation in the SWW case was, in this sense, not exceptional; for reasons such as the issues of transfers of responsibility, power, and resources (Begg 2018; Islam et al. 2018; Slavíková et al. 2019; Andráško 2021), in most states flood risk continues to be prevailingly managed via regulations or processes executed and enforced by the state agencies (Geaves and Penning-Rowsell 2015; Slavikova 2018; Matczak and Hegger 2021). Moreover, even if these authorities may have the best intentions in terms of involving other stakeholders in the planning processes, they by themselves are bounded by hierarchies of policy-making, and confusing/incoherent frameworks for action (Fekete et al. 2021); the relevance of these factors has been recognized also by the PMO’s opposition. Additionally, depending on epistemic lock-ins (Raška et al. 2022), the seeming reluctance to adopt “newer” approaches can simply result also from a lack of professional experience with them, including respective guidelines or adequate institutional frameworks (Collentine and Futter 2018; Albrecht and Hartmann 2021). It still holds though, that the SWW exemplified several aspects of the shifts within the (Czech) FRM: the MCA, a well-established method (Walczykiewicz 2015; Ruangpan et al. 2021) was used here for the first time in the CR for a large-scale flood-protection project; the polder variant was approved for construction, despite the lack of experience with such a measure (*PO: “We have several smaller polders … but this polder is the biggest”*); and despite lacking any “vehement invitations”, or providing limited options to participate, processes of relevant decision-making are now relatively open in the country. Chiefly organized groups equipped not only with enthusiasm but also professional knowledge thus have a chance to actively engage and decisively affect the respective outcomes.

Unlike other projects (Kirchherr et al. 2016; Hartmann et al. 2018; Potočki et al. 2022), relocations, flooding of residential areas, availability of land, or planned land-use changes were not reasons for local resistance in the SWW case. The municipalities lying closer to the waterwork were aware of the benefits of the SWW for downstream riparians (Kirchherr et al. 2016; Collentine and Futter 2018; Seher and Löschner 2018); thus, although reflecting on the proximity issue, no typical signs of the so-called NIMBYism (Devine-Wright 2013) were recorded. Furthermore, the main dispute was a bit obscure for the locals, who *“chiefly want … just to have it finally done” (NGO2)*. As aptly pointed out (Fekete et al. 2021), in current approaches, lay people are stereotyped as “the decision-makers”; they, by themselves though, may not be interested in taking responsibility for what will be decided. Last but not least, the locals were a bit “excluded” from the whole process. The PMO organized public meetings/hearings, yet these seemed to be there rather to inform than to engage, reminding a formality to serve some mandatory requirements in an (possibly) eyewash manner (Fekete et al. 2021). The NGOs’ initiative centred on local interests, yet also acknowledged that the inclusion of experts and politicians was the key to the success; otherwise, the voices of locals would stay muted, or simply be not effective enough.

### The “conflict”

When it comes to water-related projects, conflicting views are nothing surprising (Zolghadr-Asli et al. 2021; Raška et al. 2023). The SWW case comprised (sometimes) conflicting relations between the PMO and mayors, or differing views of the project held by locals or even higher levels of decision-making (SA1 stated that the Ministry of the Environment of the CR *“fought bravely”* for the dry polder, while Ministry of Agriculture *“pushed for”* the water reservoir/dam); the dispute between the dam proponents and opposition, however, attracted the most attention, including its media coverage *(SA2: “no other waterworks was known as much … in the CR”)*. Differing rationalities and evolving expectations in terms of pluralism in planning processes (Slavíková et al. 2019; Raška et al. 2023) manifested here clearly, yet rather than a “problem”, the situation has positive connotations. Not only did both parties stand on common grounds in terms of some major developments within the FRM (such as recognition of the importance of NBS), but also the nature of the dispute suggested a significant move from “blocked/thwarted communication” (Musil 1995) in the case of the Nové Mlýny waterworks (Table 1), to more opened, balanced, inclusive, and transparent ways of interaction, potentially enhancing the legitimacy of the decisions made (Welp et al. 2006; Begg 2018; Singh et al. 2022). To make the next step in this process, the role of a neutral agent/negotiator facilitating the participatory processes in the future has been highlighted by scholars (Reed 2008; Geaves and Penning-Rowsell 2015) but also by some of the interviewees (e.g. ACAD2). Another aspect to be considered is the continued engagement once measures have been implemented (Potočki et al. 2022); accounting for the project’s expected long-term development, only the future of the SWW project will show whether the initiative motivated by a threat to public good will continue their endeavour to maximize the efficiency and benefits of the variant they were eventually able to push through.

## Conclusion

In this study, we traced back and reconstructed the evolution of the Skalička Waterwork (SWW) project, one of the most debated and media-covered water-related, flood-protection-centred projects in the Czech Republic. Several twists and tipping points within the case have been identified, reflecting the changing preferences for particular measures, yet also the involvement of individual actors/stakeholders, and their differing views over the “right solution”; the case eventually crystallized into the “dam versus polder” dispute. The “wet variant” (i.e. an on-flow dam/water reservoir with a constant water level), proposed by the state river basin administration, has been opposed here by a joint initiative of NGOs, local activists, and politicians, expressing concerns about the “wet variant's” impacts on the local landscape, and proposing and pushing for an alternative scheme of side dry polder. The opposition succeeded not only in terms of a renewed assessment of the project, but finally, the side polder has been approved by the government to be realized. The resolution has, however, a bittersweet taste for all the parties involved: since for the majority of the dam’s opposition, any large-scale measure construction in the area was unnecessary/unwelcome, even the side polder variant was seen by them as a “compromise” or “lesser evil” needed to be done and accepted in a situation when the complete abandonment of the project was unattainable; the state river basin administrators, while now they can continue with the preparation of the project, feel also wronged and disappointed by the preference of a variant supposedly pushed through by media coverage and political pressures rather than by expertise; finally, the locals/local representatives, standing for the whole time a bit aside from the main dispute, face the uncertainty of a further postponement of the project realization and the region’s flood-protection improvement.

Compared to other water-related projects, the SWW case is in many ways specific: it did not entail large-scale relocations and flooding of residential areas, or land- or buyouts-related issues; both main parties largely relied on the views of scholars, experts, and other stakeholders; the economic aspect of the project was not prioritized; and the opposition to the state administration succeeded, ascribing the achievement to a unified, constructive initiative of diverse actors, building upon—besides the necessary political support and other circumstances—the proposal of an alternative variant and a joined local and scientific knowledge, providing for the key argumentation within the case. The case is, however, also emblematic of recent developments in the FRM: a multitude of participants were involved, including their differing/conflicting views, but also joined (local and scientific) knowledge; the issues of redefining the actors’ (mostly the state’s) roles came to the fore; the communication was not easygoing, yet, principally, democratic and mostly free of antipathies; the complex, catchment-wide perspective was applied; and the importance of adaptation to/mitigation of extreme weather/climate events was commonly acknowledged, including the increasing preference for the nature-based and hybrid solutions.
